# Urokinase-type plasminogen activator and plasminogen activator inhibitor-1 complex as a serum biomarker for COVID-19

**DOI:** 10.3389/fimmu.2023.1299792

**Published:** 2024-01-11

**Authors:** Tetiana Yatsenko, Ricardo Rios, Tatiane Nogueira, Yousef Salama, Satoshi Takahashi, Yoko Tabe, Toshio Naito, Kazuhisa Takahashi, Koichi Hattori, Beate Heissig

**Affiliations:** ^1^ Department of Research Support Utilizing Bioresource Bank, Graduate School of Medicine, Juntendo University School of Medicine, Tokyo, Japan; ^2^ Department of Enzymes Chemistry and Biochemistry, Palladin Institute of Biochemistry of the National Academy of Science of Ukraine, Kyiv, Ukraine; ^3^ Institute of Computing, Federal University of Bahia, Salvador, Bahia, Brazil; ^4^ An-Najah Center for Cancer and Stem Cell Research, Faculty of Medicine and Health Sciences, An-Najah National University, Nablus, Palestine; ^5^ Division of Clinical Precision Research Platform, the Institute of Medical Science, the University of Tokyo, Tokyo, Japan; ^6^ Department of Respiratory Medicine, Juntendo University School of Medicine, Tokyo, Japan; ^7^ Center for Genome and Regenerative Medicine, Juntendo University, Graduate School of Medicine, Tokyo, Japan; ^8^ Department of Hematology/Oncology, the Institute of Medical Science, the University of Tokyo, Tokyo, Japan

**Keywords:** COVID-19, plasminogen activator inhibitor-1, urokinase-type plasminogen activator, interleukin-6, fibrinolysis, thrombosis, C-reactive protein, respiratory distress syndrome

## Abstract

Patients with coronavirus disease-2019 (COVID-19) have an increased risk of thrombosis and acute respiratory distress syndrome (ARDS). Thrombosis is often attributed to increases in plasminogen activator inhibitor-1 (PAI-1) and a shut-down of fibrinolysis (blood clot dissolution). Decreased urokinase-type plasminogen activator (uPA), a protease necessary for cell-associated plasmin generation, and increased tissue-type plasminogen activator (tPA) and PAI-1 levels have been reported in COVID-19 patients. Because these factors can occur in free and complexed forms with differences in their biological functions, we examined the predictive impact of uPA, tPA, and PAI-1 in their free forms and complexes as a biomarker for COVID-19 severity and the development of ARDS. In this retrospective study of 69 Japanese adults hospitalized with COVID-19 and 20 healthy donors, we found elevated free, non-complexed PAI-1 antigen, low circulating uPA, and uPA/PAI-1 but not tPA/PAI-1 complex levels to be associated with COVID-19 severity and ARDS development. This biomarker profile was typical for patients in the complicated phase. Lack of PAI-1 activity in circulation despite free, non-complexed PAI-1 protein and plasmin/α2anti-plasmin complex correlated with suPAR and sVCAM levels, markers indicating endothelial dysfunction. Furthermore, uPA/PAI-1 complex levels positively correlated with TNFα, a cytokine reported to trigger inflammatory cell death and tissue damage. Those levels also positively correlated with lymphopenia and the pro-inflammatory factors interleukin1β (IL1β), IL6, and C-reactive protein, markers associated with the anti-viral inflammatory response. These findings argue for using uPA and uPA/PAI-1 as novel biomarkers to detect patients at risk of developing severe COVID-19, including ARDS.

## Highlights

- Lower circulating uPA and uPA/PAI-1 complex levels and increased non-complexed PAI-1 were associated with severe COVID-19 outcomes (ARDS).
- uPA/PAI-1 complex positively correlated with proinflammatory response (TNFα, IL1β, IL6, CRP) in severely ill patients.

## Introduction

Impaired blood clot dissolution (hypofibrinolysis) and hypercoagulation, inflammation, and endotheliopathy contribute to the pathogenesis of coronavirus disease-2019 (COVID-19) (1–6).

D-dimers, fibrin fragments generated after fibrin dissolution (7), have been proposed in various studies to predict COVID-19 disease severity. However, Sridharan et al. reported that D-dimer levels are ineffective as a prognostic marker of COVID-19-associated thrombosis (8). We showed that Japanese COVID-19 patients presented with low D-dimer levels even in the complicated phase (5). A low thrombosis incidence and fewer severe cases were reported in Japan compared to other countries globally (9). As of March 17^th,^ 2023, the death rate of COVID-19 in Japan and the Western Pacific region is 0.22% and 0.2%, respectively, compared to that in Europe (0.8%), South-East Asia (1.32%), Eastern Mediterranean region (1.5%), Americas (1.54%), and Africa (1.84%) (covid19.who.int).

The levels of other fibrinolytic factors, including tissue-type plasminogen activator (tPA), urokinase-type PA (uPA), and one of its main inhibitors called plasminogen activator inhibitor-1 (PAI-1) and the soluble form of the uPA receptor (suPAR) were found to be altered in COVID-19 patients (10). Dysregulation of uPA and its receptor system has been implicated in the pathogenesis of pulmonary fibrosis and acute respiratory distress syndrome (ARDS) (10–12). While these studies indicate that the fibrinolytic system during COVID-19 is altered, they did not consider that all these factors occur not only in their free forms but also in complexes that change their biological functions.

The plasminogen-plasmin system, best known for removing fibrin-containing blood clots, controls extracellular matrix degradation and inflammation, the latter due to its ability to activate other proteases and release pro-inflammatory cytokines (13, 14). Only a small portion of circulating plasminogen is converted to plasmin. Free plasmin in the blood plasma can be immediately inhibited by α2-antiplasmin (α2AP). Plasmin is activated on fibrin by tPA or urokinase-type plasminogen activator (uPA) after binding to receptors like uPAR on cell surfaces (15). Plasminogen activator inhibitor-1 (PAI-1) and PAI-2 can bind tPA or uPA and block their fibrinolytic activity. Inflammation promotes the local release of tPA and PAI-1 from endothelial cells (16). Elevated PAI-1 levels modulate neutrophil migration in murine models of bacterial infection or abdominal surgery (17–19). Viral infections are associated with elevated circulating PAI-1 levels (20). Intracellular endothelial PAI-1 impairs endothelial function (21).

Circulating free, relatively unstable active PAI-1 readily converts to the latent or inactive form by binding to PAs or undergoing structural changes or proteolytical cleavage (e.g., by neutrophil elastase) in less than 1h (22–24). Vitronectin binding to PAI-1 stabilizes the active form and extends the lifetime of active inhibitor (25). uPA, PAI-1, and uPA/PAI-1 complexes are important prognostic factors in metastatic breast cancer (26). The uPA/PAI-1 complex typically constitutes ~5% of the uPA antigen (total uPA), and elevated levels indicate breast cancer metastasis (27).

Tissue destruction is a common feature of COVID-19-associated ARDS. uPA is involved in the matrix degradation of tissues during inflammation (28) and tissue fibrosis (29). Studies also show a relationship between coagulopathy and systemic inflammation (30). Plasmin controls TNFα and IFNγ expression in macrophages (31, 32). TNFα and IFNγ in synergy can trigger inflammatory cell death, tissue damage, and mortality in SARS-CoV2 infection and cytokine shock syndromes (33).

Because fibrinolytic factors can occur in free and complexed forms with differences in their biological functions, we examined the predictive impact of uPA, tPA, and PAI-1 in their free forms and complexes as a prognostic biomarker for COVID-19 severity and the development of ARDS, and determined their association with inflammatory factors.

## Materials and methods

### Study design, participants, and data collection

The study population included adults of Japanese origin admitted to Juntendo University, Tokyo, Japan. All study participants gave informed consent to anonymize their clinical data. The inclusion criteria were adult patients older than 18 years, inpatients and outpatients, patients admitted to the hospital with a positive PCR or rapid antigen test for SARS-CoV-2, availability of basic medical information, including ethnicity, patient history, initial blood laboratory data, and outcome data. The study period spanned March 2020 to February 2021. Eight healthy volunteers of Japanese origin (aged 35 to 57 years) and 12 healthy volunteers of European ancestry (aged 22 to 57) were also recruited and served as controls.

We defined the clinical phases of COVID-19 at diagnosis as 1) uncomplicated (either asymptomatic or with symptoms of upper respiratory tract infection, fever, nausea, emesis, or diarrhea), 2) complicated (need for oxygen supplementation or clinically relevant increase in prior oxygen home therapy, partial pressure of oxygen (PaO2) at room air < 70 mmHg, SO2 at room air < 90%, new cardiac arrhythmia, new pericardial effusion > 1 cm or new heart failure with pulmonary edema, congestive hepatopathy, or peripheral edema), and 3) critical (need for life-supporting therapy, such as mechanical ventilation, catecholamine dependence, life-threatening cardiac arrhythmia, liver failure with an INR > 3.5, a quick sequential organ failure assessment score > + 2, or acute renal failure with the need for dialysis) (34). Criteria for discharge were the absence of fever for at least three days, substantial improvement in both lungs, clinical remission of respiratory symptoms, and one negative PCR test result for SARS-CoV2 RNA.

Heart comorbidities included patients with a history of myocardial infarction, coronary disease, and congestive heart failure. Patient blood examinations included the coagulation/fibrinolysis factors D-dimer (≤1 µg/ml), INR (<1.25), fibrinogen (≤400 mg/dl), and platelet counts (120,000–450,000/µl) as well as the inflammatory factors CRP (≤0.3 mg/dl), IL6 (≤1.8 pg/ml), WBC (4000–8000/ul), and neutrophils (2000–8900/ul). The normal range of each factor is given in brackets.

### Blood sample collection and serum preparation

Serum samples were collected after whole blood collection, clotting, and centrifugation at 400 g for 10 minutes at RT without brake. The undiluted serum was then aliquoted and stored in polypropylene tubes at −80°C for subsequent analysis.

### Quantification of PAI-1 activity

According to the manufacturer’s protocol, PAI-1 activity was measured in freshly thawed serum with Human Plasminogen Activator Inhibitor type 1 (PAI-1) Activity ELISA kit (#CSI19809A; Cell Sciences, Newburyport, USA). Latent or complexed PAI-1 will not bind to the ELISA plate or be detected by the assay.

### Western blotting

Serum levels of plasminogen, tPA, uPA, PAI-1, suPAR, α2-antiplasmin, TGFβ1, TNFα, IL1β, and sVCAM1 were measured by Western blot analysis. Blood serum was mixed with 2x Laemmli Sample Buffer (#1610737, Bio-Rad, Hercules, USA) and boiled for 1 min. Serum samples (20–70 µg proteins) were applied on 10 - 12% acrylamide gel, transferred to PVDF membrane (#IPVH00010, Millipore, Burlington, USA), blocked, and incubated overnight at 4°C with primary antibody. Membranes were then incubated with a secondary antibody conjugated with horseradish peroxidase (HRP). Detection of protein bands was performed with ECL Prime Western blotting reagent (#RPN2236, Amersham, Amersham, UK) using an image analyzer (Image-Quant LAS4000, GE Healthcare Life Sciences, Chicago, USA).

### List of antibodies used for the analysis (with working dilutions)

PAI-1 rabbit polyclonal antibodies (1:1000, #ab66705, Abcam, Cambridge, UK); tPA rabbit polyclonal antibodies (1:1000, #sc-15346, Santa Cruz, Dallas, USA); uPA rabbit polyclonal antibodies (1:1000, #17968-1-AP, Proteintech, San Diego, USA); uPAR rabbit polyclonal antibodies (1 ug/ml) (35); Plg rabbit polyclonal antibodies (0,5 ug/ml) (36); TGFβ1 rabbit polyclonal antibodies (1:1000, #sc-146, Santa Cruz, Dallas, USA); TNFα rabbit polyclonal antibodies (Cell Signaling #3707S, 1:1000); VCAM1 rabbit polyclonal antibodies (1:1000, #sc8304, Santa Cruz, Dallas, USA); α2-antiplasmin rabbit polyclonal antibodies (1:1000, #sc73659, Santa Cruz, Dallas, USA); IL1β hamster polyclonal were kindly provided by members of the FACS Core Facility of Juntendo University (1 ug/ml); secondary rabbit anti hamster HRP-conjugated (1:2000, #ab5745, Abcam, Cambridge, UK); secondary goat anti-rabbit polyclonal HRP-conjugated (1:8000, #sc2004, Santa Cruz, Dallas, USA). Quantification of the protein bands was performed with ImageJ software.

### Quantitative reverse transcriptase-polymerase chain reaction

Total RNA was extracted from peripheral mononuclear cells of patients using Trizol reagent and reverse-transcribed into complementary DNA (cDNA) using the High Capacity cDNA Reverse Transcription kit (#4368813, Applied Biosystems, Pleasanton, USA) according to the manufacturer’s instructions. cDNA was stored at –30°C. The mRNA expression levels were determined by qPCR using the QuantStudio 3 real-time PCR system (Applied Biosystems, Pleasanton, USA) with the TB Green Premix Ex Taq II (#RR820A, TaKaRa, Shiga, Japan). The relative mRNA expression was calculated using the 2-ΔΔCt method.

The respective forward and reverse primers used are shown in **Table 1**.

**Table 1:** Primer sequences used in the study.

	Forward:	Reverse:
**β-Actin**	**5`-CCAACCGCGAG** **AAGATGA -3`;**	**5`-CCAGAGGCGTA** **CAGGGATAG -3`**
**TNFα**	**5`-AGCCGCATCGC** **CGTCTCCTA-3`;**	**5`-AGCGCTGAGTCGG** **TCACCCTT -3`**

### Statistical analysis

Statistical analysis was carried out using Prizm GraphPad 8 for Windows. The Shapiro-Wilk test was performed to verify the distribution of certain variables. Student’s t-test (for variables with normal distribution) or the Mann–Whitney test (for variables without normal distribution) evaluated differences between two independent groups. The linear correlation test and determination of the Spearman coefficient were applied to estimate the association between tested variables.

For heatmap generation, all comparisons were performed using Pearson’s correlation, but the comparisons using ARDS were calculated using Point Biserial Correlation because its values are categorical (yes/no ARDS occurred).

For original data, please contact tetyanaa.yatsenko@gmail.com or heissig@juntendo.ac.jp.

## Results

### Phenotypic and clinical presentation of COVID-19 patients

Of the 69 Japanese SARS-CoV2-positive patients, 43 were defined as being in the uncomplicated phase of the disease and 26 as being in the complicated phase. No significant differences were found between the two groups for body mass index (BMI), age, gender, and COVID-19-associated comorbidities like heart disease, diabetes, and hypertension (**Table 2**).

**Table 2:** Demographic and clinical characteristics of COVID-19 patients.

	All (n=69)	Uncomplicated (n=43)	Complicated (n=26)	p-values
Age (yr)	62.0 [46.0-76.0]	63.0 (53.0-76.0)	52.5 (35.0-73.0)	0.1114
Gender (F/M)	47 (68.12%) / 22 (31.88%)	29 (67.44%) / 14 (32.56%)	18 (69.23%) / 8 (30.77%)	1
BMI (kg/m²)	23.8 [21.17-25.85]	23.4 (21.31-25.92)	23.92 (20.86-25.03)	0.8449
Heart (Yes/No)	12 (17.39%) / 57 (82.61%)	7 (16.28%) / 36 (83.72%)	5 (19.23%) / 21 (80.77%)	1
Hypertension (Yes/No)	15 (21.74%) / 54 (78.26%)	9 (20.93%) / 34 (79.07%)	6 (23.08%) / 20 (76.92%)	0.6869
Diabetes (Yes/No)	30 (43.48%) / 39 (56.52%)	20 (46.51%) / 23 (53.49%)	10 (38.46%) / 16 (61.54%)	1

Data are n (%). P-values were obtained by comparing the values by considering the clinical phase at diagnosis. p values were calculated by X2 or Fischer’s exact test.

No thrombotic events occurred in any of the 69 patients during their hospital stays. Circulating COVID-19 severity markers associated with inflammation and coagulation were tested in a univariate model at admission (5). Patients in the complicated phase had higher C-reactive protein (CRP), procalcitonin, ferritin, and interleukin 6 (IL6) levels (**Table 3**). Higher than normal troponin T levels were found independent of the disease phase. Although white blood cell counts were in the normal range, lymphocyte counts were almost two-fold lower but still in the normal range in patients in the complicated group.

**Table 3:** Clinical diagnostic markers of COVID-19 patients.

	Uncomplicated (n=43)	Complicated (n=26)	p-values
D-Dimer (0-1µg/ml)	1.4 [1.2-1.9]	2.05 [1.8-3.8]	0.0003
Fibrinogen (150-400mg/dl)	386.0 [318.0-519.25]	567.0 [464.25-647.0]	0.0017
Platelets (153-346 x10^3^/µl)	196.0 [161.5-222.5]	172.0 [142.0-203.75]	0.1372
CRP (0-0.29mg/dl)	0.25 [0.1-2.05]	5.8 [3.1-12.04]	0
WBC (3.9 x10^3^/µl)	4.8 [3.85-5.95]	4.95 [3.88-7.07]	0.6072
Ferritin (30-400ng/ml)	200.0 [90.0-463.5]	912.0 [526.0-1154.25]	0
Neutrophils (25-60 x10^2^/µl)	3.58 [2.6-1652.5]	4.22 [2.81-6.19]	0.7901
IL6 (0-4pg/ml)	3.6 [1.75-9.6]	25.3 [7.2-44.2]	0.0001
PT INR	1.02 [0.98-1.06]	1.0 [0.98-1.12]	0.5109
CK (57-240U/I)	75.0 [56.0-118.0]	88.0 [71.75-128.25]	0.3124
LDL (70-139mg/dl)	92.5 [69.75-114.5]	90.0 [73.0-111.0]	0.8716
HDL (40-70mg/dl)	44.0 [39.75-60.0]	45.0 [32.5-50.5]	0.0966
LDH (119-221U/I)	187.0 [165.0-223.5]	275.5 [238.25-337.0]	0
Procalcitonin (0-0.04ng/dl)	0.05 [0.04-0.07]	0.09 [0.06-0.17]	0.0016
Troponin T (0-0.1ng/dl)	7.5 [4.0-12.75]	12.0 [5.5-21.0]	0.2992
Lymphocytes (x10^2^/ul)	27.9 [20.0-33.25]	15.3 [11.78-21.3]	0.0003

Data are n (%). P-values were obtained by considering the clinical phase at diagnosis. p values were calculated by X2 or Fischer’s exact test.

The coagulation markers D-dimer and fibrinogen were elevated in patients in the complicated compared to the uncomplicated phase but just above the upper limit of average values, confirming recent reports (5). Coagulation-associated factors like platelet counts or prothrombin time (PT INR) in both patient groups were similar and in a normal range.

Overall, moderate activation of coagulation with a normal extrinsic pathway and mild hypofibrinolysis characterized the Japanese COVID-19 cohort.

### High PAI-1 activity in healthy Europeans compared to Japanese subjects

Hypofibrinolysis is a condition with low levels of plasminogen activators like tPA or uPA compared to plasminogen inhibitors like PAI-1. High PAI-1 levels were reported to predict COVID-19-associated thrombosis and tissue fibrosis (37). We compared blood serum PAI-1 activity of healthy Japanese (32-57 years) and European subjects (22-57 years) with normal BMI. A trend towards higher active PAI-1 levels was found in Europeans compared to Japanese healthy subjects (26.28 ± 11.68 IU/ml and 20.2 ± 5.2 IU/ml, respectively; insignificant). Serum samples of healthy Japanese subjects were used as a control group for further investigations.

### Mild fibrinolysis activation in severely ill Japanese COVID-19 patients

**Figure 1:**
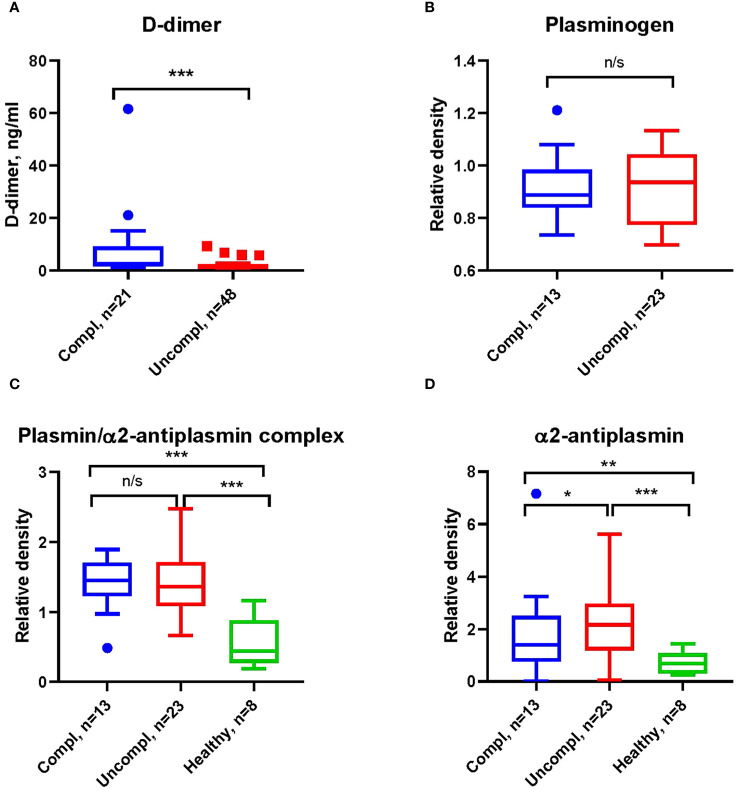
Increased fibrinolysis with increases in plasmin/α2-antiplasmin in Japanese COVID-19 patients in the complicated phase. **(A)** Levels of D-dimers of COVID-19 patients in indicated clinical phases using ELISA. **(B)** Western blot analysis for plasminogen **(B)**, plasmin/α2-antiplasmin complex **(C)**, and α2-antiplasmin **(D)** on immunoblots after loading of equal amounts of protein in serum COVID-19 patient samples. All Western blots were performed at least twice with similar results. **(B–D)** Band intensity was quantified using signal intensity and normalized to one representative sample of a healthy donor. *p < 0.1; **p < 0.05; ***p < 0.01; n/s – not significant.

COVID-19-associated endothelial damage enhances thrombus formation with fibrin deposition. Fibrin removal by plasmin generates D-dimers. D-dimer levels increased ~two-fold in COVID patients in the uncomplicated and complicated phase compared to healthy individuals (1.2-1.9 ng/ml and 1.8-3.8 ng/ml compared to <1 ng/ml) (**Figure 1A**). No difference in the plasmin precursor molecule plasminogen was found in blood samples independent of clinical phase as determined by Western blotting (**Figure 1B**).

Fibrinolysis can be regulated at the level of plasmin through α2AP (38), the primary inhibitor of plasmin in circulation. Increases in α2AP indicate hypofibrinolysis. We found higher levels of plasmin/α2AP complex and free α2AP in COVID-19 patients compared to healthy individuals (**Figures 1C, D**), indicating an increased activation of fibrinolysis with disease progression.

### Mild PAI-1 activity increases in severely ill Japanese COVID-19 patients

**Figure 2:**
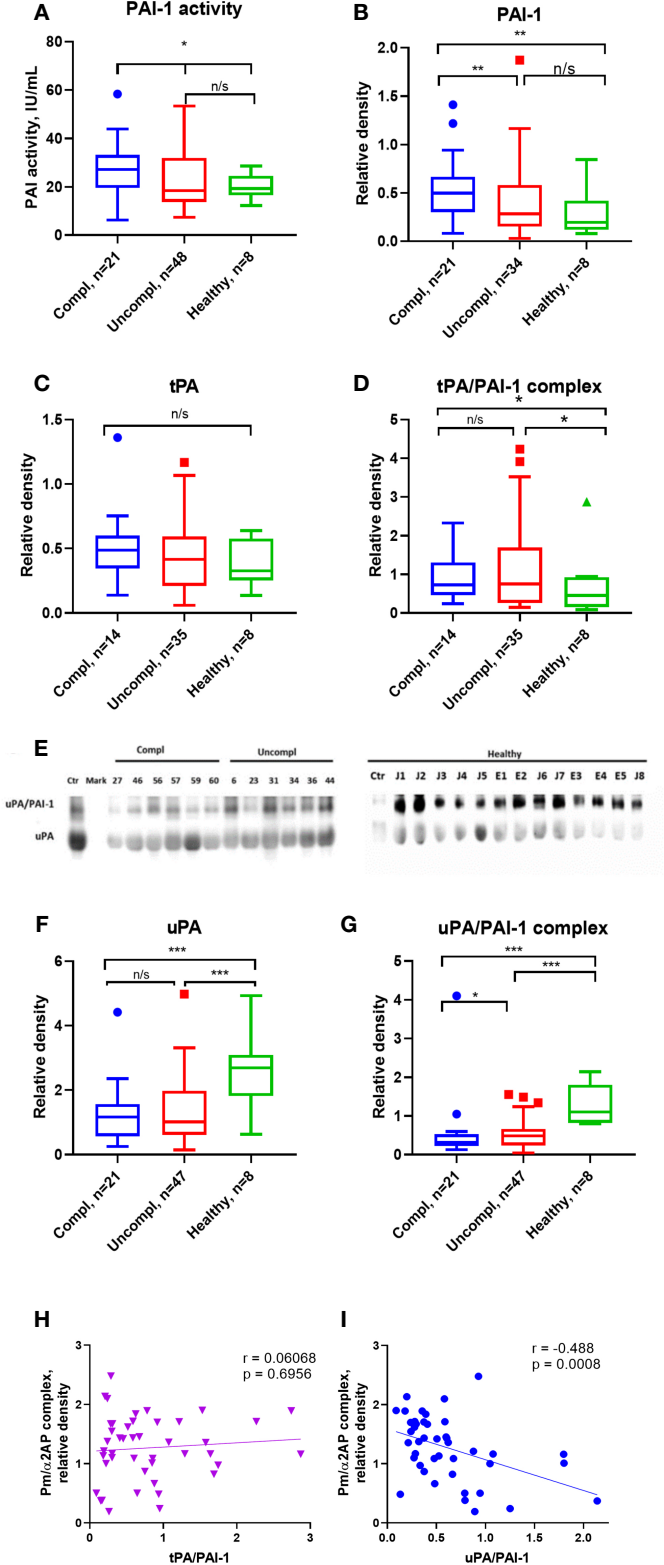
Free, non-complexed PAI-1 and uPA/PAI-1 complex levels in circulation are highest in severely ill COVID-19 patients. **(A)** Active PAI-1 (noncomplexed) levels were measured in patients and healthy donor serum using functional ELISA. Western blot analysis was used for serum level assessment of non-complexed PAI-1 antigen **(B)** with a molecular weight of 45 kDa, tPA **(C)** with a molecular weight of 70 kDa, tPA/PAI-1 complex with a molecular weight of 120 kDa **(D)**, uPA **(E, F)** with a molecular weight of 50 kDa, and uPA/PAI-1 **(E, G)** complexes detectable as 100 Da band. **(A–D, F–G)** The quantification of band intensity of single proteins or protein complexes normalized to a sample from a healthy donor is given. **(H, I)** The Spearman coefficient r was used to correlate the interrelation of PAs/PAI-1 vs. plasmin/a2-antiplasmin (n = 36 per group). *p < 0.1; **p < 0.05; ***p < 0.01; n/s – not significant.

Active PAI-1 levels were similar in sera of COVID-19 patients and healthy individuals (**Figure 2A**). Surprisingly, circulating non-complexed total PAI-1 protein (inactive and active protein) was highest in COVID-19 patients in the complicated compared to those in the uncomplicated phase and healthy donors (**Figure 2B**). Circulating free non-complexed active PAI-1 can be converted to a proteolytically inactivated free form or inactive forms after binding to tPA, forming the tPA/PAI-1 complex or binding to uPA generating the uPA/PAI-1 complexes.

tPA serum levels were similar in COVID-19 patients and healthy individuals (**Figure 2C**). TPA/PAI-1 complex serum levels were higher in COVID-19 patients, regardless of the clinical phase in healthy individuals (**Figure 2D**).

uPA serum levels were lower in COVID-19 patients than in healthy individuals, irrespective of the clinical phase (**Figures 2E, F**). Surprisingly, circulating uPA-PAI-1 protein was downregulated in COVID-19 patients compared to healthy individuals, and the lowest uPA-PAI-1 complex levels were found in patients in the complicated phase of the disease (**Figure 2G**). These data suggested that circulating PAI-1 was upregulated. In contrast, uPA and uPA/PAI-1 complexes were downregulated during disease progression.

To understand whether these PA changes reflected impaired fibrinolysis, we correlated the plasmin/α2AP complex – a good measure of actual plasmin production and fibrinolysis in circulation – with uPA/PAI-1, free uPA, and tPA/PAI-1. Plasmin/α2AP complex negatively correlated with uPA/PAI-1, but not tPA/PAI-1 complex or free uPA levels (Spearman correlation coefficient [r_s_]=-0.488, versus [r_s_]=0.0068 for tPA/PAI (**Figures 2H, I**) and [r_s_]=-0.017 for free uPA (data not shown). Only uPA/PAI-1 complexes correlated with plasmin/α2AP levels, indicating that uPA mainly generates plasmin.

### Endothelial dysfunction markers in COVID-19 are linked to fibrinolytic system activation

**Figure 3:**
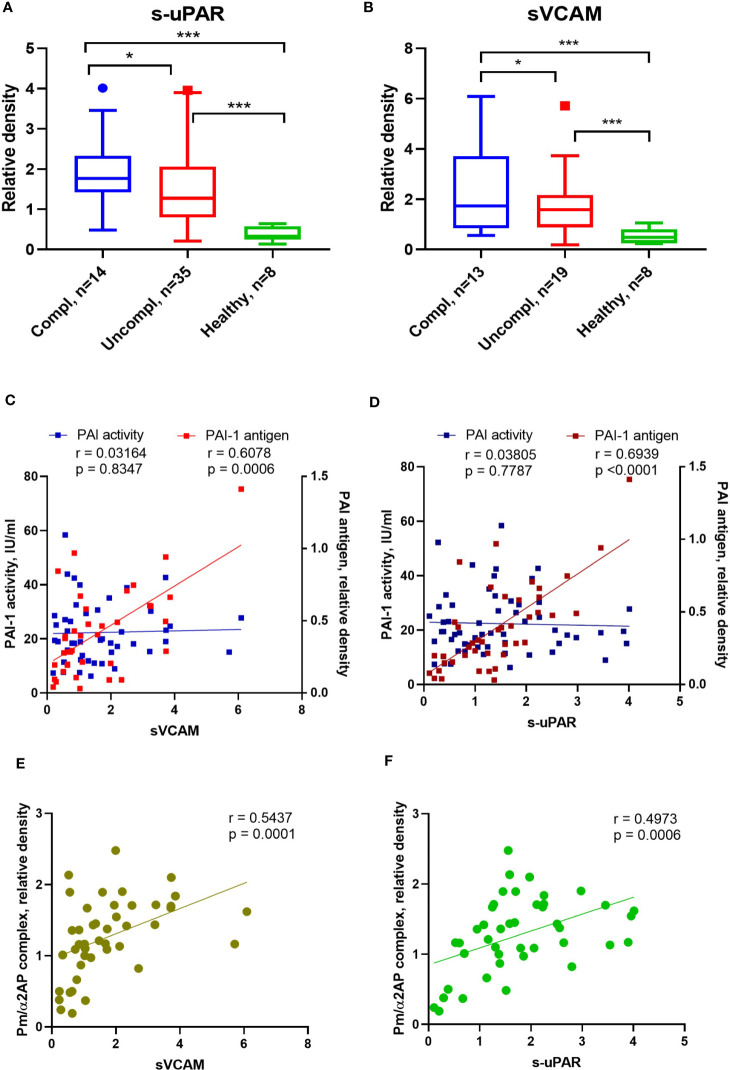
Alterations of endothelial dysfunction serum markers in COVID-19 patients related to plasmin activity. Serum soluble VCAM-1 (sVACM) **(A)** and uPAR **(B)** were measured using Western blot analysis, and the quantification of band intensity normalized to a sample from a healthy donor was given. The Spearman coefficient *r* was used for the correlation analysis of serum sVCAM1 and suPAR against PAI-1 activity (n = 45 per group) and non-complexed PAI-1 antigen (n = 36 per group) **(C, D)**. Another Spearman correlation was done for sVCAM1 and suPAR against plasmin/α2AP) (Pm/α2AP) (n = 36 per group) **(E, F)**. N depicts the number of patients tested in each group. *p < 0.1; **p < 0.05; ***p < 0.01; n/s – not significant.

SARS-CoV2 triggers endothelial dysfunction in human pulmonary microvascular endothelial cells (39). The uPAR and the endothelial-associated adhesion molecule vascular cell adhesion protein 1 (VCAM-1) are cleaved from activated endothelium by pericellular proteases, like plasmin, elastase, and matrix metalloproteinases (40, 41). Released suPAR and sVCAM1 in the serum of COVID-19 patients indicate endothelial dysfunction during COVID-19. Admission serum levels of suPAR and sVCAM1 were greater among COVID-19 patients in the uncomplicated phase compared to healthy individuals and highest in COVID-19 patients in the complicated phase (**Figures 3A, B**).

PAI-1 increases are recognized as an early marker of endothelial dysfunction. Next, we analyzed the correlation between non-complexed PAI-1 protein and PAI-1 activity or suPAR and sVCAM. Non-complexed PAI-1 protein and plasmin/α2AP complex, rather than PAI-1 activity, correlated with suPAR and sVCAM (**Figures 3C–F**). These data suggest that non-complexed PAI-1 protein and plasmin/α2AP complex are good indicators of endothelial dysfunction and that pericellular plasmin contributes to suPAR and sVCAM shedding.

### The inflammation markers interleukin1β/6, CRP, and transforming growth factor β, but not tumor necrosis factorα increase in severely ill patients

**Figure 4:**
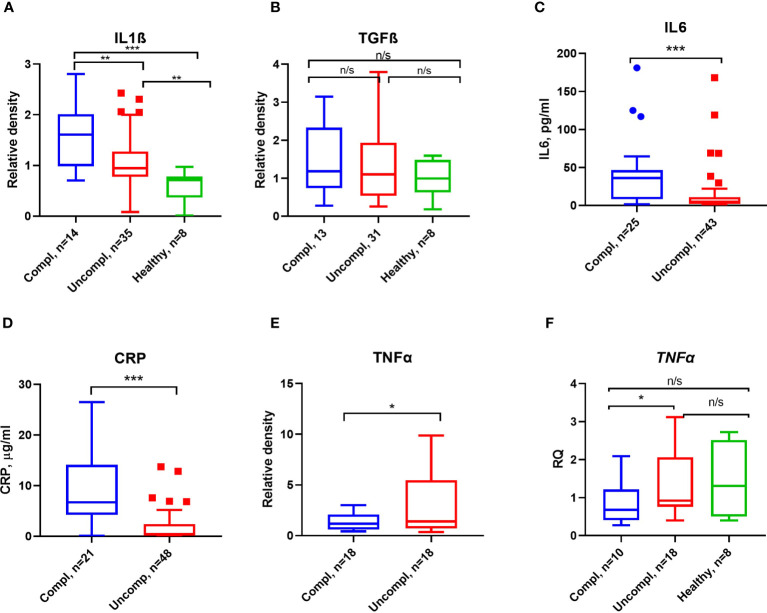
Inflammatory markers and proinflammatory cytokines in COVID-19. ELISA measured CRP **(A)** and IL6 **(B)** antigen levels. Serum IL1β **(C)**, TGFβ **(D)**, and TNFα **(E)** were detected by Western blot analysis, and quantification of band intensity normalized to a sample from a healthy donor is given. **(F)** Fold change in TNFα transcript levels determined by qPCR. Expression was normalized using the β-actin expression in the same samples. Each sample was run in triplicate. N depicts the number of patients tested in each group. *p < 0.1; **p < 0.05; ***p < 0.01; n/s – not significant.

Proinflammatory factors like interleukin1β/6, transforming growth factor β (TGFβ), and tumor necrosis factor α (TNFα) can regulate the expression of PAI-1 (42). As reported by others, CRP, IL6, IL1β, but not TGFβ levels increased in patients with more severe stages of COVID-19 (**Figures 4A–D**).

Surprisingly, low TNFα serum levels and transcripts in peripheral blood mononuclear cells (PBMCs) were found in severely ill patients compared to patients in the uncomplicated phase (**Figures 4E, F**). These data indicate that PAI-1-associated proinflammatory cytokines, TNFα excepted, were elevated in severely ill patients.

**Figure 5:**
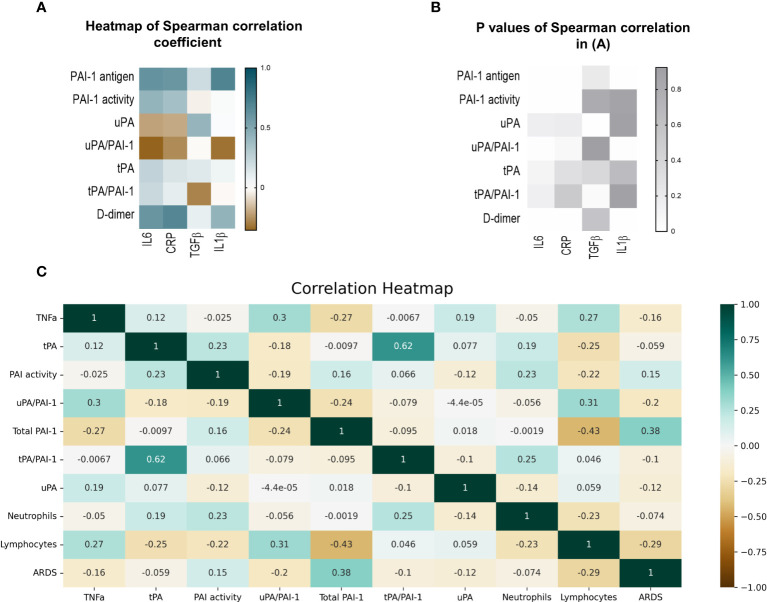
Correlation of fibrinolytic and inflammation markers in COVID-19 patients. **(A)** Heatmap of Spearman correlation coefficient. Pairs with positive coefficients are in brown, and those with negative coefficients are in blue. **(B)** P values associated with Spearman’s correlation of different parameters. **(C)** Heat map of Pearson’s correlation coefficient biomarkers of COVID-19 patients. The correlation coefficients are represented in the orange/blue color intensity change, shown in the color bar, whereby variables indicated by green or orange were positively or negatively correlated, respectively. Correlations were statistically significant (p<0.05) with 95% CIs for each correlation coefficient. **(D)** Model demonstrating the differences between COVID-19 patients in the uncomplicated (right side) and complicated (left side) phases. PAI-1, plasminogen activator inhibitor1); tPA, tissue-type plasminogen activator; uPA, urokinase plasminogen activator; SARS-CoV2, Severe acute respiratory syndrome coronavirus 2.

Spearman analysis was performed to elucidate the correlation of total PAI-1 and uPA/PAI-1 with proinflammatory cytokines. Showing a pattern similar to D-dimers, but with a stronger correlation, total PAI-1 protein positively correlated with CRP, IL6, and IL1β (Spearman coefficient r: 0,59, 0,61, 0,69; p<0,001), but not with TGFβ (r=-0.035, p=0,2; **Figures 5A, B**). The latter showed a moderate correlation with uPA (r=0,42, p=0,02). In contrast, uPA/PAI-1 negatively correlated with CRP, IL6, and IL1β. The data indicate that increases in inflammatory cytokines are linked with high total PAI-1 antigen and low uPA/PAI-1 complex levels. PAI-1 activity demonstrated a weak correlation with IL6 and CRP (r: 0.42 and 0.36; p<0,01). Our data suggest that PAI-1 in Japanese COVID-19 patients reflects the inflammatory response and is responsible for the low risk of thrombosis in these patients.

### uPA/PAI-1 and non-complexed PAI-1 correlate with ARDS

ARDS and lymphopenia are typical clinical features of severe cases of COVID-19 (43). Therefore, we tested whether the newly identified biomarkers uPA and or uPA-PAI-1 complexes might be novel biomarkers to indicate the worsening of clinical parameters such as the development of ARDS or lymphopenia even in a COVID-19 patient cohort where D-dimer levels were low. Pearson’s correlation analysis was used for numerical parameters (**Figure 5C**).

Non-complexed PAI-1 protein strongly correlated with ARDS (Point Biserial correlation analysis) and inversely with lymphocytes or TNFα. uPA/PAI-1 complex, but not tPA-PAI-1 complex, showed the opposite correlation pattern: uPA/PAI-1 complex negatively correlated with ARDS and positively with lymphocytes. TNFα positively correlated with uPA/PAI-1 complexes and negatively associated with non-complexed PAI-1 protein.

COVID-19 disease severity is often observed in patients with underlying comorbidities such as hypertension, heart disease, and diabetes. As those comorbidities also could influence the fibrinolytic response, we tested PAs and their complexes in comorbidity subgroups (**Table 4**). High uncomplexed PAI-1 levels correlated with the presence of diabetes but not heart disease or hypertension. Low uPA/PAI-1 complex levels were associated with hypertension and heart disease but not diabetes.

**Table 4:** Fibrinolytic factors of uncomplexed and complexed PAI-1 and uPA analyzed in comorbidity subgroups.

Comorbidity			
Marker	Yes	No	p-value
Hypertension			
**Patient number**	21-27	31-39	
**PAI-1 activity**	27 | 25.63 [17.66-31.37]	39 | 23.02 [14.79-31.47]	0.4263
**Uncomplexed PAI-1**	21 | 0.51 [0.26-0.67]	31 | 0.43 [0.17-0.55]	0.2712
**uPA/PAI-1 complex**	27 | 0.44 [0.24-0.58]	38 | 0.6 [0.24-0.62]	0.46
**uPA**	27 | 1.16 [0.6-1.43]	38 | 1.46 [0.57-1.99]	0.4054
Heart disease			
**Patient number**	8-10	44-56	
**PAI-1 activity**	10 | 18.16 [13.25-19.71]	56 | 25.14 [16.13-32.22]	0.1056
**Uncomplexed PAI-1**	8 | 0.43 [0.26-0.68]	44 | 0.47 [0.2-0.62]	0.9504
**uPA/PAI-1 complex**	10 | 0.35 [0.22-0.31]	55 | 0.57 [0.25-0.62]	0.0891
**uPA**	10 | 1.41 [0.68-1.6]	55 | 1.32 [0.57-1.8]	0.9203
Diabetes			
**Patient number**	11-13	41-53	
**PAI-1 activity**	13 | 28.7 [19.95-32.86]	53 | 22.95 [14.53-31.25]	0.1105
**Uncomplexed PAI-1**	11 | 0.73 [0.46-0.73]	41 | 0.39 [0.18-0.5]	0.0088
**uPA/PAI-1 complex**	13 | 0.47 [0.27-0.57]	52 | 0.56 [0.24-0.61]	0.6999
**uPA**	13 | 0.93 [0.62-1.32]	52 | 1.44 [0.58-1.99]	0.228

Data are means (data range) and p Values calculated by Mann Whitney Test in comorbidity subgroups (hypertension: yes, patients with known hypertension; no, patients without hypertension).

## Discussion

Many studies measured single factors of the fibrinolytic system, like total PAI-1, tPA, or uPA, presenting those as independent risk factors for poor outcomes in COVID-19 patients (11, 12). For example, studies reporting on PAI-1 elevation in COVID-19 ignore that although PAI-1 antigen and activity levels are correlated, antigens do not necessarily reflect PAI-1 activity levels responsible for hypofibrinolysis and fibrin persistence. Therefore, we analyzed fibrinolytic factors in their free and complexed forms and tested their value as predictors of COVID-19 severity.

Here, we identified PAI-1 in its free and uncomplexed forms with uPA as a biomarker of COVID-19 disease severity. Specifically, we show that circulating uPA, uncomplexed PAI-1, and uPA/PAI-1 complexes were differentially expressed in patients in the uncomplicated compared to the complicated phase (**Figure 5D**). We provide evidence that high total PAI-1 antigen and low uPA/PAI-1 complexes correlate with high Pm/α2AP complexes, a parameter indicating plasmin formation. Our data show that increases in inflammatory cytokines are linked with high total PAI-1 antigen and low uPA/PAI-1 complex levels. High levels of circulating non-complexed PAI-1 protein and low uPA/PAI-1 complex correlated with disease severity and ARDS, a typical and often deadly complication of severe COVID-19. Thus, this study identifies uPA and PAI-1 in their free forms and complexes as prognostic biomarkers for COVID-19 severity, the viral-associated inflammatory response, and the development of ARDS.

During clotting, a necessary requisite for generating serum, platelets are activated and release PAI-1 from its α-granules. We analyzed circulating PAI-1 and its complexes in serum samples obtained from a biobank where we could not control the collection process in the clinic. We acknowledge that the serum preparation might have contributed to the absolute PAI-1 levels measured. However, as all samples were prepared the same way, the relative changes in PAI-1 are still valuable information.

The present study on the Japanese cohort shows disease stage-dependent elevation of D‐dimer, a byproduct of fibrinolysis of cross‐linked fibrinogen, representing activation of the coagulation system and fibrinolysis. Yet, the D-dimer levels, even in the complicated group, are far below the upper limit of the normal range as found in Caucasians, as previously reported by (5). Previously, we demonstrated that Japanese COVID-19 patients in the complicated phase of the disease present with a clinical marker profile of moderate activation of coagulation, hypofibrinolysis, and impaired inflammation. In contrast, Caucasians showed a marker profile of hyperfibrinolysis and hyperinflammation (5). This study found a trend towards higher PAI-1 activity in healthy Caucasians compared to Japanese subjects, supporting earlier reports on ethnical differences in PAI-1 levels (44). The well-established lower PAI-1 levels in Japanese compared to Caucasians have been attributed to a generally lower BMI, a low percentage of patients suffering from metabolic syndrome -partially due to a plant- and seafood-based diet-, and genetic alterations of key fibrinolytic factors like tPA and PAI-1. Epidemiological studies show a low thrombosis incidence in Asians compared to African and Caucasian subgroups of the same population (45–47). Some genetic polymorphisms of coagulation system proteins may contribute to lower genetic predisposition to thrombosis in Asian ethnicities, such as lower frequencies of prothrombin, Factor V, and Factor VIII mutations (48). Still, no data link genetically determined differences in PAI-1 activity with COVID-19.

Elevated PAI-1 levels impair plasmin formation resulting in reduced fibrin degradation. Similar to our results showing high levels of circulating non-complexed PAI-1 protein, elevated PAI-1 antigen levels have been described during the SARS-CoV epidemic in 2002 (49) and the SARS-CoV-2 pandemic (50). Confirming others, we demonstrated that higher PAI-1 antigen levels were associated with a stronger inflammation response that included more circulating IL6, CRP, and IL1β, but not TGFβ levels (51, 52). Inflammatory cells, in turn, can release proinflammatory cytokines like IL6 or TNFα, which are strong stimulators of PAI-1 expression (53, 54). Surprisingly, we found that PAI-1 activity was not associated with the inflammatory response typical for COVID-19.

High levels of tPA and PAI-1 proteins were associated with worse respiratory status in COVID-19 patients (50). In contrast, no free tPA protein level increase was found in our tested Japanese COVID-19 cohort compared to a healthy control group. For the first time, we reported higher tPA/PAI-1 complexes in COVID-19 patients independent of the disease status. The data’s functional consequences are unclear, but tPA/PAI-1 complex increases have been linked to myocardial infarction (55).

While total PAI-1 protein and tPA/PAI-1 complex levels were increased, patients in the complicated phase had nearly average active PAI-1 values (56). This fibrinolytic status, in combination with high tPA/PAI-1 and plasmin/α2AP complexes, suggests a low risk of thrombosis for Japanese. Indeed, none of the study subjects experienced thrombosis, perhaps partly due to thromboprophylaxis, which has reduced Japan’s thrombosis rate from 13.5% to 1.9% (57). Even absent such prophylaxis, the thrombosis incidence in COVID-19 patients from Japan is several-fold lower than those reported by Western countries, like the Netherlands (13.5 vs. 40.8%) (2, 57–59).

The two-chain uPA has high profibrinolytic activity on the cell surface and, in its free form, is promptly and irreversibly inhibited by active PAI-1 (60). Because the secreted single-chain uPA proenzyme has low affinity to PAI-1 and low plasminogen-plasmin conversion activity, circulating free uPA is not a good marker of plasminogen activation potential and therefore did not correlate with the plasmin/α2AP complex. Only uPA/PAI-1 complexes correlated with plasmin/α2AP levels, indicating that plasmin is mainly generated by uPA.

uPA downregulation, as found in autopsies of COVID-19 lungs, impairs plasmin-driven fibrin degradation and resolution of established clots reflected in low D-dimer formation (61–64). We report that circulating uPA and uPA/PAI-1 complexes negatively correlate with ARDS. Our data are corroborated by others showing uPA and uPAR downregulation in bronchoalveolar lavage fluid of COVID-19 patients compared to healthy controls (64). uPA/PAI-1 impairs macrophage migration locally (65) and TNFα release from macrophages (66). Further studies will be required to understand how uPA/PAI-1 alters macrophage inflammatory response.

Elevated PAI-1 levels are a typical feature of metabolic syndrome, which includes clinical conditions such as obesity, type 2 diabetes, hypertension, and elevated triglyceride [reviewed in (16)]. Indeed, most of those metabolic syndrome-associated abnormalities are risk factors for severe COVID-19. We found that higher uncomplexed PAI-1 was associated with diabetes. Our clinical study cannot discern whether low uPA/PAI-1 complex or high uncomplexed PAI-1 protein levels are due to viral infection or preexisting comorbidity in patients. Controlled animal studies or prospective clinical studies should be undertaken to understand whether comorbidities and/or the SARS-CoV2 infection are responsible for decreasing uPA/PAI-1 and increasing uncomplexed PAI-1.

Plasmin-mediated releases of suPAR and sVCAM1 from endothelial cells and PAI-1 increases are indicators of COVID-19-associated endothelial dysfunction (16, 67, 68). We found increased suPAR, sVCAM1, non-complexed PAI-1 protein, and plasmin/α2AP complex levels correlated with COVID-19 severity.

We showed that the uPA/PAI-1 complex positively correlated with TNFα expression in mononuclear cells from COVID-19 patients. TNFα contributes to organ damage and predicts poor outcomes in COVID-19 patients (33). A decreased TNFα production in Japanese COVID-19 patients may contribute to a less severe disease course.

Future studies will be necessary to understand the functional consequences of the uPA/PAI-1 and tPA/PAI-1 complex formation in COVID-19 and inflammation. Our data argue that the analysis of uPA, uPA/PAI-1 complex, and non-complexed PAI-1 should be included in the fibrinolysis status of COVID-19 patients, as they can identify “fibrinolysis-sleeper/shutdown” patients with low D-dimer levels who progress rapidly into the complicated phase. Significant decreases in circulating uPA and uPA/PAI-1 complex levels may be a novel biomarker of COVID-19 severity.

## Data availability statement

The raw data supporting the conclusions of this article will be made available by the authors, without undue reservation.

## Ethics statement

All human subjects gave freely their informed consent to participate in the study. The ethics committee of Juntendo University (reference no 19-113, M20-0094) approved the study. The studies were conducted in accordance with the local legislation and institutional requirements. The participants provided their written informed consent to participate in this study. Written informed consent was obtained from the individual(s) for the publication of any potentially identifiable images or data included in this article.

## Author contributions

BH: Conceptualization, Funding acquisition, Writing – original draft. RR: Formal analysis, Methodology, Software, Writing – review & editing. TNo: Formal analysis, Methodology, Software, Writing – review & editing. YS: Data curation, Writing – review & editing. ST: Funding acquisition, Writing – review & editing. YT: Resources, Writing – review & editing. TNa: Resources, Writing – review & editing. KT: Resources, Writing – review & editing. KH: Conceptualization, Funding acquisition, Writing – review & editing. TY: Conceptualization, Data curation, Formal analysis, Investigation, Methodology, Visualization, Writing – review & editing, Writing – original draft.
