# Corrigendum: Urokinase-type plasminogen activator and plasminogen activator inhibitor-1 complex as a serum biomarker for COVID-19

**DOI:** 10.3389/fimmu.2024.1390698

**Published:** 2024-03-13

**Authors:** Tetiana Yatsenko, Ricardo Rios, Tatiane Nogueira, Yousef Salama, Satoshi Takahashi, Yoko Tabe, Toshio Naito, Kazuhisa Takahashi, Koichi Hattori, Beate Heissig

**Affiliations:** ^1^ Department of Research Support Utilizing Bioresource Bank, Graduate School of Medicine, Juntendo University School of Medicine, Tokyo, Japan; ^2^ Department of Enzymes Chemistry and Biochemistry, Palladin Institute of Biochemistry of the National Academy of Science of Ukraine, Kyiv, Ukraine; ^3^ Institute of Computing, Federal University of Bahia, Salvador, Bahia, Brazil; ^4^ An-Najah Center for Cancer and Stem Cell Research, Faculty of Medicine and Health Sciences, An-Najah National University, Nablus, Palestine; ^5^ Division of Clinical Precision Research Platform, the Institute of Medical Science, the University of Tokyo, Tokyo, Japan; ^6^ Department of Respiratory Medicine, Juntendo University School of Medicine, Tokyo, Japan; ^7^ Center for Genome and Regenerative Medicine, Juntendo University, Graduate School of Medicine, Tokyo, Japan; ^8^ Department of Hematology/Oncology, the Institute of Medical Science, the University of Tokyo, Tokyo, Japan

**Keywords:** COVID-19, plasminogen activator inhibitor-1, urokinase-type plasminogen activator, interleukin-6, fibrinolysis, thrombosis, C-reactive protein, respiratory distress syndrome

In the published article, there was an error in the author list, and author Yousef Salama, along with their affiliation, was erroneously excluded. The corrected author list and affiliations appear below.

Tetiana Yatsenko1,2, Ricardo Rios3, Tatiane Nogueira3, Yousef Salama4, Satoshi Takahashi5, Yoko Tabe1, Toshio Naito1, Kazuhisa Takahashi1,6, Koichi Hattori 7,8* and Beate Heissig 1*

1Department of Research Support Utilizing Bioresource Bank, Graduate School of Medicine, Juntendo University School of Medicine, Tokyo, Japan

2Department of Enzymes Chemistry and Biochemistry, Palladin Institute of Biochemistry of the National Academy of Science of Ukraine, Kyiv, Ukraine

3Institute of Computing, Federal University of Bahia, Salvador, Bahia, Brazil

4 An-Najah Center for Cancer and Stem Cell Research, Faculty of Medicine and Health Sciences, An-Najah National University, Nablus, Palestine;

5Division of Clinical Precision Research Platform, the Institute of Medical Science, the University of Tokyo, Tokyo, Japan

6Department of Respiratory Medicine, Juntendo University School of Medicine, Tokyo, Japan

7Center for Genome and Regenerative Medicine, Juntendo University, Graduate School of Medicine, Tokyo, Japan

8Department of Hematology/Oncology, the Institute of Medical Science, the University of Tokyo, Tokyo, Japan

The authors apologize for these errors and state that they do not change the scientific conclusions of the article in any way. The original article has been updated.

In the published article, there was an error in the **Author Contribution** section. Due to the fact author Yousef Salama was erroneously excluded, their contributions were not included in this section. The corrected **Author Contribution** section appears below.

**Author contributions**

BH: Conceptualization, Funding acquisition, Writing – original draft. RR: Formal analysis, Methodology, Software, Writing – review & editing. TNo: Formal analysis, Methodology, Software, Writing – review & editing. YS: Data curation, Writing – review & editing. ST: Funding acquisition, Writing – review & editing. YT: Resources, Writing – review & editing. TNa: Resources, Writing – review & editing. KT: Resources, Writing – review & editing. KH: Conceptualization, Funding acquisition, Writing – review & editing. TY: Conceptualization, Data curation, Formal analysis, Investigation, Methodology, Visualization, Writing – review & editing, Writing – original draft.

The authors apologize for this error and state that this does not change the scientific conclusions of the article in any way. The original article has been updated.

